# The effect of adding nanodiamond and calcium carbonate on flexural strength of resin modified and conventional glass ionomer

**DOI:** 10.4317/jced.63027

**Published:** 2025-09-01

**Authors:** Farahnaz Sharafeddin, Maryam Jamshidi, Marzieh Moradian

**Affiliations:** 1 Professor, Department of Operative Dentistry, Biomaterials Research Center, School of Dentistry, Shiraz University of Medical Sciences, Shiraz, Iran; 2 Post-graduate student, Department of Operative Dentistry, School of Dentistry, Shiraz University of Medical Sciences, Shiraz, Iran; 3 Assistant Professor, Department of Operative Dentistry, School of Dentistry, Shiraz University of Medical Sciences, Shiraz, Iran

## Abstract

**Background:**

This *in vitro* study evaluated the effect of adding two types of nanoparticles—nanodiamonds and calcium carbonate—to conventional glass ionomer cement (GIC) and resin-modified glass ionomer cement (RMGIC) on their flexural strength.

**Material and Methods:**

Using a precision digital scale, 0.2 wt.% nanodiamond particles or 4 wt.% calcium carbonate nanoparticles were added to the powders of CGIC (GC Fuji II LC Gold, GC Corp., Japan) and RMGIC (GC Fuji IX LC Gold; GC Corp., Japan). Six groups of materials were prepared (*n*=10 each): 1) GIC, 2) GIC with 0.2 wt.% nanodiamond, 3) GIC with 4 wt.% calcium carbonate, 4) RMGIC, 5) RMGIC with 0.2 wt.% nanodiamond, and 6) RMGIC with 4 wt.% calcium carbonate. The mixtures were placed into rectangular molds (25 mm × 2 mm × 2 mm), and flexural strength was measured using a universal testing machine. Data were analyzed using the Shapiro–Wilk test, two-way ANOVA, and Tukey’s post hoc test (α = 0.05).

**Results:**

Group 6 (RMGIC + 4% calcium carbonate) showed the highest flexural strength (31.90 MPa) among all groups (*P* < 0.001). The flexural strength of the CGIC group (7.96 MPa) was significantly lower than that of all other groups (*P* < 0.001), except for Group 2 (GIC + 0.2% nanodiamonds). The flexural strengths of Groups 2 and 3 (GIC + 4% calcium carbonate) were statistically similar. RMGIC groups exhibited significantly higher flexural strength compared to their corresponding CGIC groups (*P* < 0.05).

**Conclusions:**

Adding 4 wt.% calcium carbonate to both GIC and RMGIC enhances their resistance and clinical performance in stress-bearing areas. The addition of 0.2 wt.% nanodiamonds improved the flexural strength of RMGIC, although to a lesser extent than calcium carbonate.

** Key words:**Glass ionomer, Resin modified glass ionomer, Nanodiamonds, Calcium Carbonate, Flexural Strength.

## Introduction

Glass ionomer cement (GIC) is recognized as a restorative material used in various clinical contexts, including the restoration of primary teeth, Class III and Class V cavities, and non-cavitated lesions [1]. Its distinct chemistry sets GIC apart from other restorative materials, allowing it to bond effectively to both enamel and dentin while protecting against decay at the margins of restorations through fluoride release [2,3]. Additionally, GIC exhibits a low thermal expansion coefficient, offers satisfactory aesthetics, and is relatively easy to apply [4]. Despite these advantages, GICs are not widely used as permanent restorative materials in stress-bearing areas due to their low fracture toughness, tensile strength, wear resistance, and hardness [5]. To enhance its clinical performance, resin was incorporated into the formulation, resulting in improved physical and mechanical properties of conventional GIC. The resulting material, resin-modified GIC (RMGIC), undergoes light-activated polymerization, offering benefits such as extended working time, improved control over the setting process, enhanced aesthetics, and reduced water sorption [6].

Various materials, including fibers, nanoparticles, and zirconia, have been employed to improve the mechanical properties of GICs. More recently, nanoscale fillers have been introduced to further enhance GIC properties [1,3]. Nanotechnology involves creating materials with dimensions smaller than 100 nm. GICs combined with nanostructures exhibit fewer air voids and microcracks, improved handling characteristics, and increased compressive strength [7].

The addition of carbon-based nanoparticles, such as nano-graphene oxide, has also been shown to enhance the shear bond strength of both GIC and RMGIC [1]. Among these, nanodiamonds have been investigated for dental applications due to their favorable surface chemistry [8,9]. Incorporating nanodiamonds into GICs has been shown to increase the release of aluminum, silicon, strontium, and sodium ions [10]. However, the effects of adding nanodiamonds on the physico-mechanical properties of GICs and RMGICs have not yet been evaluated.

Calcium carbonate nanoparticles are well-regarded for their low toxicity, excellent biocompatibility, biodegradability, and osteoconductivity [11]. They have been shown to enhance the mechanical and thermal properties of polymers [12,13]. Incorporating calcium carbonate nanoparticles into ceramic composites improves flexural strength, fracture toughness, and resistance to micro-crack propagation [14]. In dentistry, adding calcium carbonate nanoparticles has been found to improve the mechanical resistance and compressive strength of Portland cement, although it reduces the compressive strength of mineral trioxide aggregate (MTA) [15]. However, no prior studies have examined the effect of adding calcium carbonate nanoparticles to GIC and RMGIC on their physical properties.

Therefore, the present *in vitro* study aimed to evaluate the effect of adding 0.2 wt.% nanodiamond and 4 wt.% calcium carbonate nanoparticles on the flexural strength of GIC and RMGIC. The null hypothesis of this study was that the addition of these nanoparticles would not improve the flexural strength of CGIC or RMGIC.

## Material and Methods

The protocol of this experimental study was approved by the Ethics Committee of Shiraz University of Medical Sciences (Code: IR.SUMS.DENTAL.REC.1403.007). To prepare the samples, nanoparticles were first incorporated into the GIC and RMGIC powders. Using a precision digital scale (A&D, GR+360, Tokyo, Japan; accuracy: 0.0001 g), 0.2 wt.% nanodiamond particles (3–10 nm diameter, 272.62 m²/g surface area; US-NANO, Houston, TX, USA) or 4 wt.% calcium carbonate nanoparticles (15–40 nm diameter, 30–60 m²/g surface area; American Elements, Los Angeles, CA, USA) were added to the powders of CGIC (GC Fuji II LC Gold, GC Corp., Japan) and RMGIC (GC Fuji IX LC Gold; GC Corp., Japan). The powders and nanoparticles were initially hand-mixed and homogenized using a vibrator. To prevent aggregation, the mixture was sieved. The blended powders were then weighed and transferred into clean, empty amalgam capsules, followed by mixing in an amalgamator (Ultramat 2; SDI, Australia) for 20 seconds to promote uniform distribution, as described in previous studies [16,17].

- Sample preparation

The sample size was determined to be 10 specimens per group, based on the expectation of detecting a minimum effect size of f = 0.5 in the ANOVA test, with a Type I error level of 0.05 and a test power of 80%. A total of six groups were prepared (n = 10 each):

• Group 1: CGIC 

• Group 2: CGIC with 0.2 wt.% nanodiamond

• Group 3: CGIC with 4 wt.% calcium carbonate

• Group 4: RMGIC 

• Group 5: RMGIC with 0.2 wt.% nanodiamond

• Group 6: RMGIC with 4 wt.% calcium carbonate

For each mold, GIC samples were prepared by mixing one scoop of powder (with or without nanoparticles) with one drop of the corresponding liquid, according to the manufacturer’s instructions. For RMGIC, one scoop of powder (with or without nanoparticles) was mixed with two drops of liquid. Mixing was performed manually using a plastic spatula on a cooled glass slab for 25 seconds.

**Figure 1:**
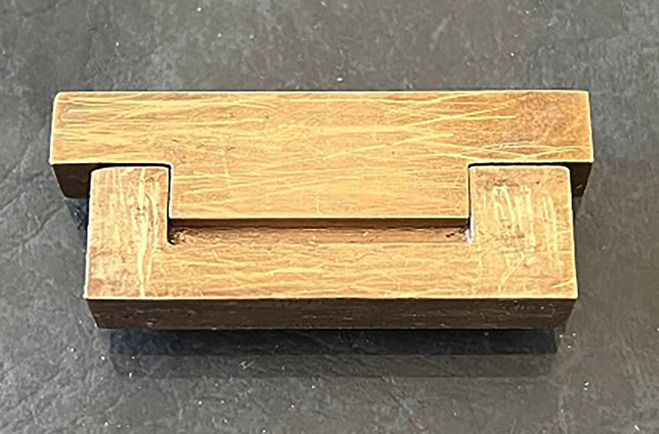
Brass mold used to prepare the samples.

The mixtures were packed into rectangular brass molds (25 mm × 2 mm × 2 mm) using a composite placement instrument (Fig. 1). A transparent polyester matrix strip (Fintrec, M-TP; Pulpdent Corp., USA) was placed over the mold during setting.

**Figure 2:**
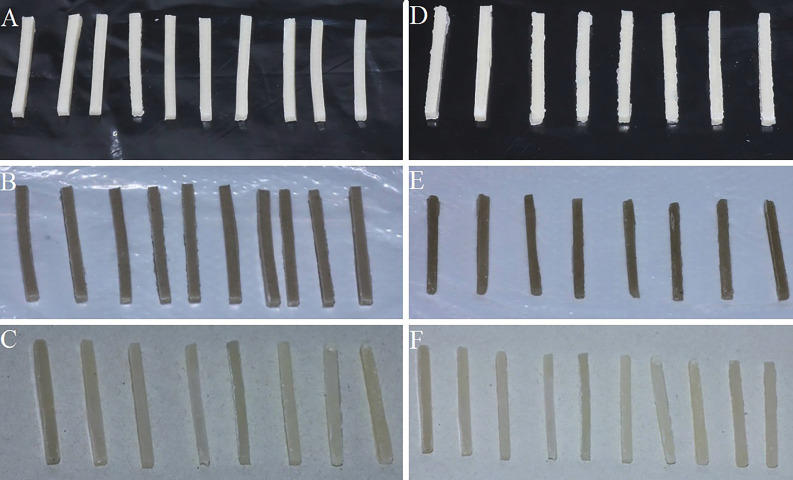
A) CGIC, B) CGIC with 0.2 wt.% nanodiamond, C) CGIC with 4 wt.% calcium carbonate, D) RMGIC, E) RMGIC with 0.2 wt.% nanodiamond, F) RMGIC with 4 wt.% calcium carbonate.

CGIC samples were allowed to set chemically for 5.5 minutes. RMGIC samples were light-cured for 20 seconds using an LED curing unit (BlueLEX; Monitex, Taiwan; intensity: 1200 mW/cm², tip diameter: 8 mm) at three overlapping points, maintaining a 1 mm distance from the surface. After setting, all specimens were removed from the molds (Fig. 2) and coated with a protective varnish (GC Corp., Tokyo, Japan) to shield them from moisture [17]. Specimens were then stored in an incubator (Nuve, Turkey) at 37 °C and approximately 100% humidity for 24 hours prior to testing. All samples were prepared by the same researcher to ensure consistency.

- Flexural strength measurement

Data analysis was conducted using SPSS version 22 (SPSS Inc., IL, USA). The normality of the data was assessed using the Shapiro–Wilk test. Two-way ANOVA followed by Tukey’s post hoc test was employed to compare the flexural strength among the groups. A *p-value* less than 0.05 was considered statistically significant.

- Statistical analysis

Data analysis was performed using SPSS version 22 (SPSS Inc., IL, USA). Normality was assessed via the Shapiro–Wilk test. Two-way ANOVA and Tukey’s post hoc test were used to compare the flexural strength between the groups. To ensure the adequacy of the sample size, the effect size was calculated. A *p-value* lower than 0.05 was considered statistically significant.

## Results

The calculated effect size was large (Cohen’s d = 0.992), confirming that the sample size was sufficient. The mean flexural strength for all groups is presented in  Table 1. The Shapiro–Wilk test confirmed that the data followed a normal distribution (*P* > 0.05). Two-way ANOVA revealed a statistically significant difference in flexural strength among all groups (*P* < 0.001).

Post hoc analysis using Tukey’s test indicated that Group 6 (RMGIC + 4% calcium carbonate) exhibited the highest flexural strength (31.90 MPa), significantly outperforming all other groups (*P* < 0.001).

The CGIC control group (7.96 MPa) demonstrated significantly lower flexural strength compared to all other groups (*P* < 0.001), except Group 2 (CGIC + 0.2% nanodiamond), which showed no statistically significant difference. The flexural strengths of Groups 2 and 3 (CGIC + 4% calcium carbonate) were statistically similar. Overall, the RMGIC-based groups showed significantly higher flexural strength than their corresponding CGIC groups ( Table 1).

## Discussion

The present study evaluated the effect of adding 0.2 wt.% nanodiamonds and 4 wt.% calcium carbonate on the flexural strength of GIC and RMGIC. Flexural strength is widely recognized as a key indicator of material resistance and durability in clinical settings [18]. Enhancing the mechanical properties of GICs broadens their clinical applications, particularly for permanent restorations in posterior teeth [19].

This study found that incorporating 4 wt.% calcium carbonate enhanced the flexural strength of both GIC and RMGIC. Notably, RMGIC with 4 wt.% calcium carbonate exhibited the highest flexural strength among all tested groups. Calcium carbonate is known to improve the mechanical properties of dental materials by filling microcracks in cementitious substances and inhibiting crack propagation [20]. It also acts as a nucleation site that accelerates the hydration process, promoting the formation of calcium-silicate-hydrate (C–S–H) gels [21]. As the C–S–H gel matures, it fills voids, interlocks with residual crystalline phases, and enhances the overall mechanical performance [22].

Other research has similarly reported the benefits of adding calcium carbonate to dental materials. For instance, He *et al*. [23] demonstrated that adding 4 wt.% nano calcium carbonate to epoxy resin resulted in good dispersion and significantly improved thermal stability, compressive strength, flexural strength, and impact strength, which aligns with our findings. However, increasing the concentration to 8 wt.% resulted in reduced mechanical properties due to particle agglomeration. Uneven nanoparticle distribution may also contribute to crack formation and porosity in the cement [24]. Furthermore, incorporating carbonate ions into hydroxyapatite has been shown to enhance mechanical strength and fracture toughness [25]. Ismail *et al*. [26] also reported that adding 1.0 wt.% calcium carbonate to zirconia-toughened alumina ceramics increased surface microhardness and fracture toughness. These findings support the effectiveness of small amounts of calcium carbonate in enhancing the mechanical properties of GIC and RMGIC.

Some studies have used calcium carbonate-rich materials, such as chicken eggshells and seashells, rather than pure calcium carbonate. Allam *et al*. [27] found that incorporating 3% chicken eggshell powder into GIC significantly enhanced compressive strength. Similarly, Albasso *et al*. [28] reported that adding 10% seashells to GIC improved compressive strength, microhardness, and fluoride release. Although these studies did not use calcium carbonate nanoparticles specifically, their findings align with ours in demonstrating the positive impact of calcium carbonate on the mechanical strength of GICs.

Adding 0.2% nanodiamonds to RMGIC increased its flexural strength. Nanodiamonds possess exceptional strength, chemical stability, and thermal conductivity [29]. To our knowledge, this is the first study to incorporate nanodiamonds into GICs. The enhancement in RMGIC may be due to the uniform dispersion of nanodiamonds within the resin matrix, which facilitates better stress distribution from the matrix to the fillers [30]. Other studies have also reported improvements in mechanical properties with nanodiamond additions. For example, incorporating 0.25%–0.5% nanodiamonds into heat-polymerized acrylic resin significantly enhanced flexural strength and elastic modulus [29,31]. Mangal *et al*. [32] found that adding 0.1–0.5 wt.% nanodiamonds to autopolymerized PMMA improved flexural strength, elastic modulus, and surface hardness. Chu *et al*. similarly reported a considerable increase in flexural strength and elastic modulus when 0.2% nanodiamonds were added to composite resin. These positive findings support the current study’s conclusion regarding the benefits of nanodiamonds in RMGIC.

In contrast, the addition of nanodiamonds did not increase the flexural strength of GIC. This difference is likely due to the absence of a resin matrix in GIC. Nanodiamond particles form strong covalent bonds with resin molecules, enhancing matrix recovery after deformation and contributing to the reinforcement effect [33]. The selected nanodiamond concentration was based on previous studies on PMMA [29,31,32] and composite resin [34], where increasing concentrations tended to reduce mechanical performance. Although Chu *et al*. [34] used a similar 0.2% concentration, the materials differ significantly from GIC and RMGIC in composition and application. Therefore, further research is needed to determine the optimal nanodiamond concentration specifically for GIC and RMGIC.

The present study also found that RMGIC with calcium carbonate had significantly higher flexural strength than RMGIC containing nanodiamonds. Conversely, GIC samples with calcium carbonate showed flexural strength comparable to those with nanodiamonds. This variation may be attributed to calcium carbonate’s interaction with hydroxyl groups in the resin matrix via its carbonyl or hydroxyl groups, which enhances the mechanical properties more in RMGIC than in GIC [35].

Furthermore, RMGIC exhibited significantly greater flexural strength than GIC, consistent with previous studies [36]. This difference may be due to the presence of hydroxyethyl methacrylate (HEMA), a hydrophilic monomer in RMGIC that improves wettability and toughness, leading to stronger micromechanical and chemical bonding to dentin [37].

The present study faced some limitations. Replicating oral conditions, such as chewing forces, thermal changes, salivary interactions, and chemical challenges from acidic foods, is difficult in laboratory settings. Moreover, only one concentration of each nanoparticle was tested. Future studies should examine different powder-to-liquid ratios in nanoparticle-modified GIC and RMGIC and evaluate the impact on bond strength, biocompatibility with oral tissues and fluoride release, which is critical for preventing dental caries formation and progression [38]. Clinical studies are also needed to confirm the long-term effectiveness of these nanoparticle-enhanced materials.

## Conclusions

The addition of 4 wt.% calcium carbonate significantly enhanced the flexural strength of both GIC and RMGIC. Incorporation of 0.2 wt.% nanodiamond improved the flexural strength of RMGIC, though to a lesser extent than calcium carbonate. Regardless of nanoparticle incorporation, RMGIC samples consistently demonstrated higher flexural strength than their corresponding GIC counterparts. Based on these findings, RMGIC reinforced with 4 wt.% calcium carbonate may be considered a promising restorative material for use in stress-bearing areas.

## Figures and Tables

**Table 1:** Mean± standard deviation of flexural strength (MPa) of GIC and RMGIC without nanoparticles, and containing nanodiamonds and calcium carbonate nanoparticles.

Groups	Flexural strength (MPa) Mean± SD	P value*
GIC	7.96±0.79a	<0.001
GIC + 0.2% nanodiamond	8.54±0.82ab	
GIC + 4% calcium carbonate	9.25±0.57b	
RMGIC	21.72±1.23c	
RMGIC + 0.2% nanodiamond	26.59±0.75d	
RMGIC + 4% calcium carbonate	31.90±1.14e	

CGIC: conventional glass ionomer cement, RMGIC: resin modified glass ionomer cement. *Two-way ANOVA result. The mean values of the different letters were statistically significant according to Tukey post-hoc test).

## Data Availability

The datasets used and/or analyzed during the current study are available from the corresponding author.

## References

[1] Ghodrati P, Sharafeddin F (2023). Evaluation of the effect of nano-graphene oxide on shear bond strength of conventional and resin-modified glass ionomer cement. Clin Exp Dent Res.

[2] Asadi M, Majidinia S, Bagheri H, Hoseinzadeh M (2024). The Effect of Formulated Dentin Remineralizing Gel Containing Hydroxyapatite, Fluoride, and Bioactive Glass on Dentin Microhardness: An In Vitro Study. Int J Dent.

[3] Sharafeddin F, Alavi A A, Siabani S, Safari M (2020). Comparison of Shear Bond Strength of Three Types of Glass Ionomer Cements Containing Hydroxyapatite Nanoparticles to Deep and Superficial Dentin. J Dent (Shiraz).

[4] Sidhu S K, Nicholson J W (2016). A review of glass-ionomer cements for clinical dentistry. J Funct Biomater.

[5] Chau N P, Pandit S, Cai J N, Lee M H, Jeon J G (2015). Relationship between fluoride release rate and anti-cariogenic biofilm activity of glass ionomer cements. Dent Mater.

[6] Agha A, Parker S, Patel M P (2016). Development of experimental resin modified glass ionomer cements (RMGICs) with reduced water uptake and dimensional change. Dent Mater.

[7] Gjorgievska E, Van Tendeloo G, Nicholson JW, Coleman NJ, Slipper IJ, Booth S (2015). The incorporation of nanoparticles into conventional glass-ionomer dental restorative cements. Microsc Microanal Microstruct.

[8] Najeeb S, Khurshid Z, Agwan AS, Zafar MS, Alrahabi M, Qasim SB (2016). Dental applications of nanodiamonds. Sci Adv Mater.

[9] Osswald S, Yushin G, Mochalin V, Kucheyev S O, Gogotsi Y (2006). Control of sp2/sp3 carbon ratio and surface chemistry of nanodiamond powders by selective oxidation in air. JACS.

[10] Krueger A, Lang D (2012). Functionality is key: recent progress in the surface modification of nanodiamond. Adv Funct Mater.

[11] Mydin RBS, Mydin RBSMN, Nadhirah I, Ishak NN, Shaida N (2018). Potential of Calcium Carbonate Nanoparticles for Therapeutic Applications. MJMHS.

[12] Parhizkar M, Shelesh-Nezhad K, Rezaei A (2016). Mechanical and thermal properties of Homo-PP/GF/CaCO3 hybrid nanocomposites. Adv Mater Res.

[13] Yang G, Heo YJ, Park SJ (2019). Effect of morphology of calcium carbonate on toughness behavior and thermal stability of epoxy-based composites. Processes.

[14] Hakamy A (2020). Effect of CaCO3 nanoparticles on the microstructure and fracture toughness of ceramic nanocomposites. J Taibah Univ Sci.

[15] Bernardi A, et al (2017). Effects of the addition of nanoparticulate calcium carbonate on setting time, dimensional change, compressive strength, solubility and pH of MTA. Int Endod J.

[16] Mahmoud N, Metwally A (2021). Fluoride release and recharging ability of glass ionomer cement incorporating hydroxyapatite nanoparticles. Egypt Dent J.

[17] Sharafeddin F, Alavi A A, Siabani S, Safari M (2020). Comparison of shear bond strength of three types of glass ionomer cements containing hydroxyapatite nanoparticles to deep and superficial dentin. J Dent.

[18] Sideridou I D, Karabela M M, Bikiaris D N (2007). Aging studies of light cured dimethacrylate-based dental resins and a resin composite in water or ethanol/water. Dent Mater.

[19] Moheet I A, et al (2019). Modifications of Glass Ionomer Cement Powder by Addition of Recently Fabricated Nano-Fillers and Their Effect on the Properties: A Review. Eur J Dent.

[20] Wang K, Zheng M, Yan S, Gao Z, Hu Y, Peng L (2024). Study on the influence mechanism of calcium carbonate particles on mechanical properties of microcrack cement. Constr Build Mater.

[21] Wu Z, Khayat K H, Shi C, Tutikian B F, Chen Q (2021). Mechanisms underlying the strength enhancement of UHPC modified with nano-SiO2 and nano-CaCO3. Cem Concr Compos.

[22] Parisay I, Moodi M, Boskabady M, Bagheri H, Salari R, Hoseinzadeh M (2025). Physical and drug- releasing properties of a cement containing simvastatin (SimCeram). BMC Oral Health.

[23] He H, Li K, Wang J, Sun G, Li Y, Wang J (2011). Study on thermal and mechanical properties of nano-calcium carbonate/epoxy composites. Mater Design.

[24] Mortazavi V, Fathi M, Ataei E, Khodaeian N, Askari N (2012). Shear bond strengths and morphological evaluation of filled and unfilled adhesive interfaces to enamel and dentine. Int J Dent.

[25] Bhatnagar D, Gautam S, Batra H, Goyal N (2023). Enhancement of Fracture Toughness in carbonate doped Hydroxyapatite based nanocomposites: Rietveld analysis and Mechanical behaviour. J Mech Behav Biomed Mater.

[26] Ismail H, Mohamad H (2023). Effects of CaCO3 additive on the phase, physical, mechanical, and microstructural properties of zirconia-toughened alumina-CeO2-Nb2O5 ceramics. Ceram Int.

[27] Allam G, Abd El-Geleel O (2018). Evaluating the Mechanical Properties, and Calcium and Fluoride Release of Glass-Ionomer Cement Modified with Chicken Eggshell Powder. Dent J.

[28] Albasso A S, Ali R R, Yahya A A (2024). In vitro evaluation of some mechanical properties and fluoride release of glass-ionomer cement modified with seashell nanoparticles. J Dent Res Dent Clin Dent Prospects.

[29] Fouda S M, Gad MM, Ellakany P, Al Ghamdi MA, Khan SQ, Akhtar S (2022). Flexural Properties, Impact Strength, and Hardness of Nanodiamond-Modified PMMA Denture Base Resin. Int J Biomater.

[30] Cao W, Zhang Y, Wang X, Li Q, Xiao Y, Li P (2018). Novel resin-based dental material with anti-biofilm activity and improved mechanical property by incorporating hydrophilic cationic copolymer functionalized nanodiamond. J Mater Sci Mater Med.

[31] Gad M M, Ali MS, Al-Thobity AM, Al-Dulaijan YA, El Zayat M, Emam ANM (2022). Polymethylmethacrylate Incorporating Nanodiamonds for Denture Repair: In Vitro Study on the Mechanical Properties. Eur J Dent.

[32] Mangal U, Kim JY, Seo JY, Kwon JS, Choi SH (2019). Novel Poly(Methyl Methacrylate) Containing Nanodiamond to Improve the Mechanical Properties and Fungal Resistance. Materials.

[33] Wang M, Zhang K, Hou D, Wang P (2020). Microscopic insight into nanodiamond polymer composites: reinforcement, structural, and interaction properties. Nanoscale.

[34] Chu YQ, Tong Y, Zhang TL, Huang FL (2012). Mechanical properties of dental composite resins containing nanodiamond of different diameters. BIT.

[35] He H, Zhang Z, Wang J, Li K (2013). Compressive properties of nano-calcium carbonate/epoxy and its fibre composites. Composites Part B: Engineering.

[36] Malhotra S, Bhullar KK, Kaur S, Malhotra M, Kaur R, Handa A (2022). Comparative evaluation of compressive strength and flexural strength of gc gold hybrid, gic conventional and resin-modified glass-ionomer cement. Pharm Bioallied Sci.

[37] Techa-Ungkul C, Sakoolnamarka R (2021). The effect of dentin age on the microshear bond strength and microleakage of glass-ionomer cements. Gerodontology.

[38] Parisay I, Boskabady M, Bagheri H, Babazadeh S, Hoseinzadeh M, Esmaeilzadeh F (2024). Investigating the efficacy of a varnish containing gallic acid on remineralization of enamel lesions: an in vitro study. BMC Oral Health.

